# Characterization of Proteoform Post-Translational Modifications by Top-Down and Bottom-Up Mass Spectrometry in Conjunction with Annotations

**DOI:** 10.1021/acs.jproteome.3c00207

**Published:** 2023-09-20

**Authors:** Wenrong Chen, Zhengming Ding, Yong Zang, Xiaowen Liu

**Affiliations:** † Department of BioHealth Informatics, Indiana University-Purdue University Indianapolis, Indianapolis, Indiana 46202, United States; ‡ Department of Computer Science, Tulane School of Science and Engineering, Tulane University, New Orleans, Louisiana 70118, United States; § Department of Biostatics and Health Data Sciences, Indiana University School of Medicine, Indianapolis, Indiana 46202, United States; ∥ Center for Computational Biology and Bioinformatics, Indiana University School of Medicine, Indianapolis, Indiana 46202, United States; ⊥ Tulane Center for Biomedical Informatics and Genomics, Tulane University, New Orleans, Louisiana 70112, United States; # Deming Department of Medicine, Tulane University, New Orleans, Louisiana 70112, United States

**Keywords:** post-translational modification, top-down mass spectrometry, bottom-up mass spectrometry

## Abstract

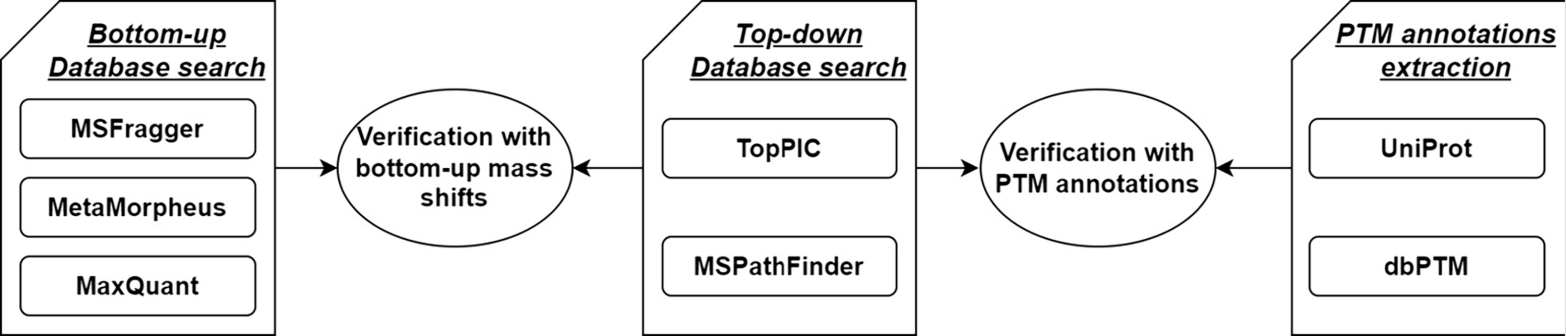

Many proteoforms can be produced from a gene due to genetic mutations, alternative splicing, post-translational modifications (PTMs), and other variations. PTMs in proteoforms play critical roles in cell signaling, protein degradation, and other biological processes. Mass spectrometry (MS) is the primary technique for investigating PTMs in proteoforms, and two alternative MS approaches, top-down and bottom-up, have complementary strengths. The combination of the two approaches has the potential to increase the sensitivity and accuracy in PTM identification and characterization. In addition, protein and PTM knowledge bases, such as UniProt, provide valuable information for PTM characterization and verification. Here, we present a software pipeline PTM-TBA (PTM characterization by Top-down and Bottom-up MS and Annotations) for identifying and localizing PTMs in proteoforms by integrating top-down and bottom-up MS as well as PTM annotations. We assessed PTM-TBA using a technical triplicate of bottom-up and top-down MS data of SW480 cells. On average, database search of the top-down MS data identified 2000 mass shifts, 814.5 (40.7%) of which were matched to 11 common PTMs and 423 of which were localized. Of the mass shifts identified by top-down MS, PTM-TBA verified 435 mass shifts using the bottom-up MS data and UniProt annotations.

## 1 Introduction

Post-translational modifications (PTMs) in proteoforms, like methylation, acetylation, and phosphorylation, play crucial roles in biological systems and diseases.^1,2^ For example, kinase phosphorylation is essential for signal transduction in cells and the development of cancer cells.^3^ PTM characterization, which identifies and localizes PTMs in proteoforms, is important for studying protein functions, understanding biological mechanisms, discovering disease biomarkers, and designing personalized vaccines.^2,4−7^

The dominant techniques for identifying PTMs in proteoforms are two complementary mass spectrometry (MS) approaches: bottom-up and top-down MS.^5,8,9^ In bottom-up MS,^5^ proteoforms are proteolytically digested into short peptides, which are separated by liquid chromatography (LC) or other separation methods and then analyzed by MS. Bottom-up MS usually provides high fragment ion coverage of identified peptides, which increases the accuracy in determining PTM types and locations in peptides. However, bottom-up MS may identify only several peptides of a protein and miss many peptides with PTMs in proteome-wide analyses.^10^ Also, PTM combinatorial patterns in proteoforms are lost in digestion, making bottom-up MS inefficient for analyzing complex proteoforms with multiple PTMs.^11^ Top-down MS analyzes intact proteins instead of peptides,^12^ so it can identify PTM combinatorial patterns in proteoforms and characterize proteoforms with multiple PTMs. But top-down MS still suffers from limited sensitivity and throughput and often fails to identify low-abundance proteoforms in proteome-wide studies. Top-down mass spectra also tend to miss many fragment ions, resulting in ambiguous localization sites for PTMs.^13^

Continuous efforts have been made to develop computational methods for identifying and localizing PTMs using bottom-up MS.^14−18^ These methods identify PTMs in peptides by database search with prespecified variable PTMs^14,16,17^ or open search.^10,15,18,19^ While a database search with variable PTMs reports the types of identified PTMs directly, the open search method first identifies unexpected mass shifts, which are then matched to common PTMs to identify their PTM types. These PTMs are further localized to determine their modification sites.^20^ Many software tools, such as MaxQuant,^14^ determine the site of a PTM in an identified peptide-spectrum-match (PSM) based on the similarity scores between the spectrum and all candidate forms of the peptide with the PTM on different sites. The modified peptide form with the best similarity score is reported as the PTM localization result. The confidence scores of PTM characterization or localization results are computed using probabilistic or machine learning models, such as AScore,^20^ SLoMo,^21^ PhosphoRS,^22^ and Andromeda score.^23^ Similarly, in top-down MS, PTMs are identified by database search with variable PTMs,^24,25^ the open search strategy,^26,27^ or spectral alignment.^27^ Confidence scores of PTM characterization and localization results are reported using Bayesian or other statistical models.^26,28,29^

Combining bottom-up and top-down MS is a promising direction for PTM identification and characterization because the two approaches have complementary strengths.^11,30^ While top-down mass spectra offer PTM combinatorial pattern information for characterizing complex proteoforms, bottom-up mass spectra provide high fragment ion coverage that top-down mass spectra often lack. Bottom-up MS can also confirm PTMs identified by top-down MS. Several existing tools, like Proteoform Suite,^30^ adopt this method for PTM characterization.

Protein and PTM knowledge bases offer additional evidence for validating PTMs identified by MS.^31^ Protein annotations in UniProt^32^ contain many PTMs reported in the literature. Additionally, dbPTM,^33^ SysPTM,^34^ and PRISMOID^35^ store both structural and functional information about PTMs.

Here, we present PTM-TBA, a software pipeline for proteoform PTM characterization by combining top-down MS, bottom-up MS, and PTM annotations. We performed a systematic evaluation of the pipeline using a technical triplicate of top-down and bottom-up MS data of SW480 colorectal cancer cells. Using top-down MS, on average, we identified 2000 mass shifts in SW480 proteoforms, of which 814.5 (40.7%) were matched to 11 high-frequency PTMs and 423 were confidently localized. PTM-TBA also verified 435 mass shifts/PTMs using peptides identified by bottom-up MS data and UniProt annotations. We also evaluated PTM-TBA on a top-down and bottom-up MS data set of Jurkat cells. Using top-down MS, we identified 1044 mass shifts, of which 372 were matched to high-frequency PTMs and 247 were localized. In addition, 168 mass shifts identified by top-down MS were confirmed by bottom-up MS and PTM annotations.

## 2 Methods

### 2.1 Data Sets

Bottom-up and top-down MS data of SW480 colorectal cancer cells^36,37^ and Jurkat cells^38^ were used to evaluate the proposed PTM characterization and validating pipeline. The bottom-up and top-down MS experiments of SW480 cells were performed in technical triplicates. In the bottom-up MS experiments,^36^ proteins of SW480 cells were digested using trypsin. The dried peptides were fractionated into five fractions using 8, 15, 22, 30, and 50% acetonitrile (ACN) in 10 nM TEAB (pH 9). A Waters NanoAcquity LC system with a BEH C18 column (Waters, 10 cm × 100 mm, 1.7 μm particle size) coupled with a Q-Exactive mass spectrometer (Thermo Fisher Scientific) was used for LC-MS analysis. The sample peptides in each fraction were separated over a 90 min linear gradient (A, 0.1% formic acid in water; B, 0.1% formic acid in ACN). The gradient was used for the samples with solvent B added from 2 to 80% from 0 to 80 min and re-equilibrated at 2% from 80 to 90 min. MS1 scans were collected from 350 to 2000 *m*/*z* at a resolution of 70,000 (at 200 *m*/*z*) with an AGC target of 1 × 10^6^ ions, and MS/MS scans were collected from 100 to 1500 *m*/*z* at a resolution of 17,500 (at 200 *m*/*z*) with an AGC target of 5 × 10^5^ ions. The top 12 precursor ions in each MS1 spectrum were isolated with a 2 *m*/*z* window for MS/MS analyses, and the normalized collision energy was set to 28. A total of 15 runs (3 replicates × 5 fractions) of bottom-up MS data were collected for SW480 cells.

The top-down MS data of SW480 cells were generated using size exclusion chromatography (SEC)-capillary zone electrophoresis (CZE)-MS/MS.^37,39^ The sample proteins were initially separated into 6 fractions using a SEC column, and each fraction was then injected into a fused silica capillary with a linear polyacrylamide (LPA) coating and a background electrolyte of 5% acetic acid for a 100 min separation. The electrospray voltage was set between 2.2 and 2.3 kV, and the separation voltage was 30 kV. The CZE system was connected to a Q-Exactive HF mass spectrometer (Thermo Fisher Scientific) for MS/MS analysis. The resolution of the MS1 and MS/MS spectra was 120,000 at 200 *m*/*z*. Using higher-energy C-trap dissociation (HCD) MS/MS, the top 5 precursor ions in each MS1 spectra were fragmented. Three technical replicates with a total of 18 runs (6 fractions × 3 replicates) were obtained for SW480 cells.

The bottom-up and top-down data sets^38^ of Jurkat cells were downloaded from MassIVE (https://MassIVE.ucsd.edu; bottom-up data set ID: MSV000083304; top-down data set ID: MSV000083768). In the bottom-up MS experiments, Jurkat cell protein was digested by trypsin and then separated into 10 fractions using high-pH reversed-phase liquid chromatography (RPLC). Each fraction was dehydrated, resuspended in a water solution of 5% ACN and 1% formic acid, and then analyzed with an HPLC-MS/MS system (nanoAcquity, Waters and LTQ Velos Orbitrap, Thermo Fisher Scientific) using a 20 cm reverse-phase capillary column packed with 3 μm C18 beads with 0.2% formic acid in water for mobile phase A and 0.2% formic acid in ACN for mobile phase B. Full MS1 scans from 300 to 1500 *m*/*z* were collected at a resolution of 60,000 (at 200 *m*/*z*). These MS1 scans were followed by top 10 precursor HCD fragmentation to produce the MS/MS spectra at a resolution of 7500 (at 200 *m*/*z*) using data-dependent acquisition (DDA). Dynamic exclusion with 60 s was activated.

In top-down MS analysis, protein mixtures extracted from Jurkat cells were separated via GELFREE into 11 fractions. Each fraction was separated by an HPLC system (nanoAcquity, Waters) with a 100 × 365 μm fused silica capillary microcolumn packed with 20 cm of 5 μm diameter, 1000 Å pore size PLRP-S resin (Agilent) for a 97 min gradient and then analyzed by a QE-HF Orbitrap mass spectrometer (Thermo Fisher Scientific). MS1 scans were conducted in the range of 500 to 1600 *m*/*z* at a resolution of 240,000 (at 200 *m*/*z*). A process of averaging 7 microscans was performed, with an AGC target of 1 × 10^6^ and a maximum injection time of 100 ms. The top three most intense peaks in the MS1 scan with a charge greater than 2 were selected for fragmentation via HCD, with a normalized collision energy setting of 25. The resolution of MS/MS scans was set to 120,000 (at 200 *m*/*z*), while the isolation window was set to 4 *m*/*z*, and the averaging process for 3 microscans was chosen. Dynamic exclusion was enabled with a duration of 30 s. Two technical replicates were generated. Because only one replicate of Jurkat bottom-up MS data was available, only the first technical replicate of the Jurkat top-down MS data was used in this study.

### 2.2 Bottom-Up MS Database Search

All raw files were converted to centroided mzML files using msconvert in ProteoWizard.^40^ A human proteome database, GENCODE-SWISS (18,417 proteins), was built based on the protein sequences shared by the GENCODE basic annotation (version 38, 19,652 proteins)^41^ and the reviewed Swiss-Prot protein sequences in the UniProt human proteome database (version 11/23/2019, 20,350 proteins)^32^ using TopPG (version 1.0).^42^ It contained a reference protein sequence for each gene that is included in both Swiss-Prot and the GENCODE basic annotation.

MSFragger^15^ (version 3.4), MetaMorpheus^43^ (version 1.0), and MaxQuant^14^ (version 2.0.3) were used separately for bottom-up mass spectral identification by database search. Two rounds of database searches were performed using MSFragger, in which the bottom-up mass spectra were searched against the GENCODE-SWISS database concatenated with a reversed decoy database (18,417 entries) using the open search strategy. The variable PTMs were set to methionine oxidation and N-terminal acetylation (NTA) in the first round and NTA only in the second round. In both rounds, cysteine carbamidomethylation was set as the fixed modification. Up to 3 variable modifications were allowed in each identified peptide, and the default setting [−150 Da, 500 Da] was used for the allowed mass shift of precursor masses. Identified PSMs were filtered using 1% spectrum-level false discovery rate (FDR). Then, the reported PSMs were grouped to obtain peptide identifications, which were filtered using a 1% peptide-level FDR. Finally, identified proteins were filtered with 1% protein-level FDR. Detailed parameter settings of MSFragger can be found in Table S1.

In MetaMorpheus, the database search function with spectral calibration and the G-PTM-D^43^ workflow for PTM discovery were utilized for peptide and PTM identification. The GENCODE-SWISS protein database was used and augmented by integrating 36 predefined PTMs (Table S2). Decoy sequences were generated by reversing the target sequences. Other parameters were set as follows: the maximum number of missed cleavages was 2; the minimum and maximum peptide lengths were 5 and 50, respectively; the initiator methionine behavior was variable; the fixed PTM was cysteine carbamidomethylation. The 36 predefined PTMs were downloaded from the GitHub repository of MetaMorpheus.^43^ Peptide identifications with mass shifts or PTMs were filtered with a *Q*-value cutoff of 0.01. Detailed parameter settings of MetaMorpheus can be found in Table S3.

Using MaxQuant^14^ (version 2.0.3), three rounds of database searches were performed. In the three rounds, the variable PTMs were set to NTA only, NTA and oxidation on methionine, and NTA and phosphorylation. In the three rounds, cysteine carbamidomethylation was set as the fixed PTM; the maximum mass of detected peptides was 5000 Da, and the fragment mass error tolerance was 20 ppm. The first search peptide mass tolerance was 20 ppm, and the main search peptide mass tolerance was 7 ppm. The maximum number of missed cleavages allowed per peptide was 2 with trypsin as the digestion enzyme. The data analysis with a target-decoy approach was conducted by using a reversed decoy database, and identifications were filtered with 1% FDR at the peptide and protein levels. Detailed parameter settings of MaxQuant can be found in Table S4.

### 2.3 Top-Down MS Database Search

The TopPIC^27^ (version 1.6.0) and MSPathFinder^24^ (version 1.1) pipelines were employed to identify proteoforms and mass shifts from top-down MS data. All raw files were converted to centroided mzML files using msconvert in ProteoWizard.^40^ In the TopPIC pipeline, TopFD (version 1.6.0)^44^ was used to deconvolute the centroided top-down mass spectra to neutral monoisotopic masses of precursor and fragment ions. The deconvoluted MS/MS spectra were searched against the GENCODE-SWISS database concatenated with a decoy database with the same size using TopPIC (version 1.6.0).^27^ An error tolerance of 10 ppm was set for precursor and fragment masses; at most, one unexpected mass shift was allowed in each identified proteoform, and cysteine carbamidomethylation was set as the fixed modification. Oxidation and phosphorylation were set as variable PTMs, and at most, one variable PTM is allowed in each identified proteoform. Identified proteoforms were filtered with 1% proteoform-level FDR. Detailed parameter settings of TopFD and TopPIC can be found in the Supporting Information, Tables S5 and S6.

In the MSPathFinder pipeline, ProMex^24^ (version 1.1) was employed to deconvolute precursor and fragment ions in the centroided mass spectra. Then, the deconvoluted precursor and fragment masses were searched against the GENCODE-SWISS protein database for proteoform identification using MSPathFinder (version 1.1). An error tolerance of 10 ppm was set for precursor and fragment masses, and a single internal cleavage of proteoforms was allowed. Cysteine carbamidomethylation was set as the fixed modification, while NTA, oxidation, and phosphorylation were set as dynamic modifications. At most, one dynamic modification was allowed per sequence. Detailed parameter settings of ProMex and MSPathFinder can be found in the Supporting Information, Tables S7 and S8.

### 2.4 Matching Mass Shifts Identified in Proteoforms and Peptides

For top-down MS data, the input of PTM-TBA is text (tsv) files containing proteoform identifications reported by TopPIC or MSPathFinder. For bottom-up MS, the input of PTM-TBA is text (tsv) files containing peptide identifications reported by MSFragger, MetaMorpheus, or MaxQuant.

Open search-based spectral identification often reports proteoforms or peptide forms with mass shifts whose modification sites cannot be confidently localized (Figure 2). A mass shift and its possible sites in a peptide/proteoform are represented by a quadruple [*m*, *p*, *a*, *b*], where *m* is the mass shift, *p* is the protein containing it, and positions *a* and *b* specify a region [*a*, *b*] of the protein that contains potential modification sites of the mass shift. Two mass shifts with the same shift and from the same protein but different sequence regions are matched if they can be explained by one modification site. Matched mass shifts are used to remove possible duplicated shifts and to find top-down and bottom-up mass shift pairs that can be explained by the same modification site.

In top-down MS, the region [*a*, *b*] of a mass shift reported by TopPIC was slightly extended to correct for possible errors in [*a*, *b*] introduced by randomly matched fragment masses. Specifically, position *a* was extended to the left until the extended part contained 2 matched fragment ions or the N-terminus was reached, and position *b* was extended to the right similarly. When the PTM type of mass shift is known, the mass shift with its PTM type is represented by a quintuple [*m*, *p*, *a*, b, and *t*], where *t* is the type of the PTM.

A mass shift [*m*_1_, *p*_1_, *a*_1_, *b*_1_] is matched to another mass shift [*m*_2_, *p*_2_, *a*_2_, *b*_2_] if (1) *p*_1_ and *p*_2_ and [*a*_2_, *b*_2_] overlap, and (3) the minimum difference among the mass pairs (*m*_1_, *m*_2_), (*m*_1_, *m*_2_ – 1.00235), and (*m*_1_, *m*_2_ + 1.00235) is smaller than an error tolerance (0.1 Da in the experiments). The mass difference of 1.00235 Da is allowed in the comparison because it is a common error in deconvoluted precursor masses in top-down MS. A mass shift with its PTM type [*m*_1_, *p*_1_, *a*_1_, *b*_1_, *t*_1_] is matched to another mass shift with its PTM type [*m*_2_, *p*_2_, *a*_2_, *b*_2_, *t*_2_] if (1) the mass shift [*m*_1_, *p*_1_, *a*_1_, *b*_1_] is matched to [*m*_2_, *p*_2_, *a*_2_, *b*_2_], (2) *t*_1_ and *t*_2_ are the same, and (3) the overlapping region of [*a*_1_, *b*_1_] and [*a*_2_, *b*_2_] contains at least one amino acid residue that can be modified by the PTM.

### 2.5 Removing Duplicated Mass Shifts

To remove duplicated mass shifts, we initially grouped the mass shifts reported from top-down or bottom-up MS data into clusters and then removed duplicated mass shifts in each cluster. In the clustering step, two mass shifts [*m*_1_, *p*_1_, *a*_1_, *b*_1_] and [*m*_2_, *p*_2_, *a*_2_, *b*_2_] are added to the same cluster if *p*_1_ and *p*_2_ are the same and the difference between *m*_1_ and *m*_2_ is smaller than an error tolerance (0.1 Da in the experiments). To remove duplicated mass shifts in a cluster, the mass shifts in the cluster are ranked using the left boundary (position *a* in the quadruple representation [*m*, *p*, *a*, *b*]) of the range containing possible modification sites. Next, we iteratively check the mass shifts in the cluster following their ranks to remove duplicated ones using a greedy algorithm (Figure S1).

### 2.6 PTMs in Protein Annotations

PTM annotations were extracted from UniProt^32^ and dbPTM.^45^ An annotation file of the UniProt human proteome (version 06/16/2022, 204, 906 entries) was downloaded from UniProt.^32^ Annotations for acetylation, hydroxylation, methylation, oxidation, and phosphorylation were downloaded from dbPTM^45^ (version 05/30/2023). PTMs and their sites in proteins were extracted from the annotation files by using Python scripts in PTM-TBA. A PTM annotation is matched to a mass shift [*m*, *p*, *a*, *b*] in a proteoform if the annotated PTM is in the region [*a*, *b*] of protein *p* and the mass shift of the PTM is matched to the mass shift *m* with an error tolerance (0.1 Da in the experiment). The error ±1.00235 Da is also allowed in the matching of the mass shift.

## 3 Results

### 3.1 Overview of the PTM-TBA Pipeline

**Figure 1:**
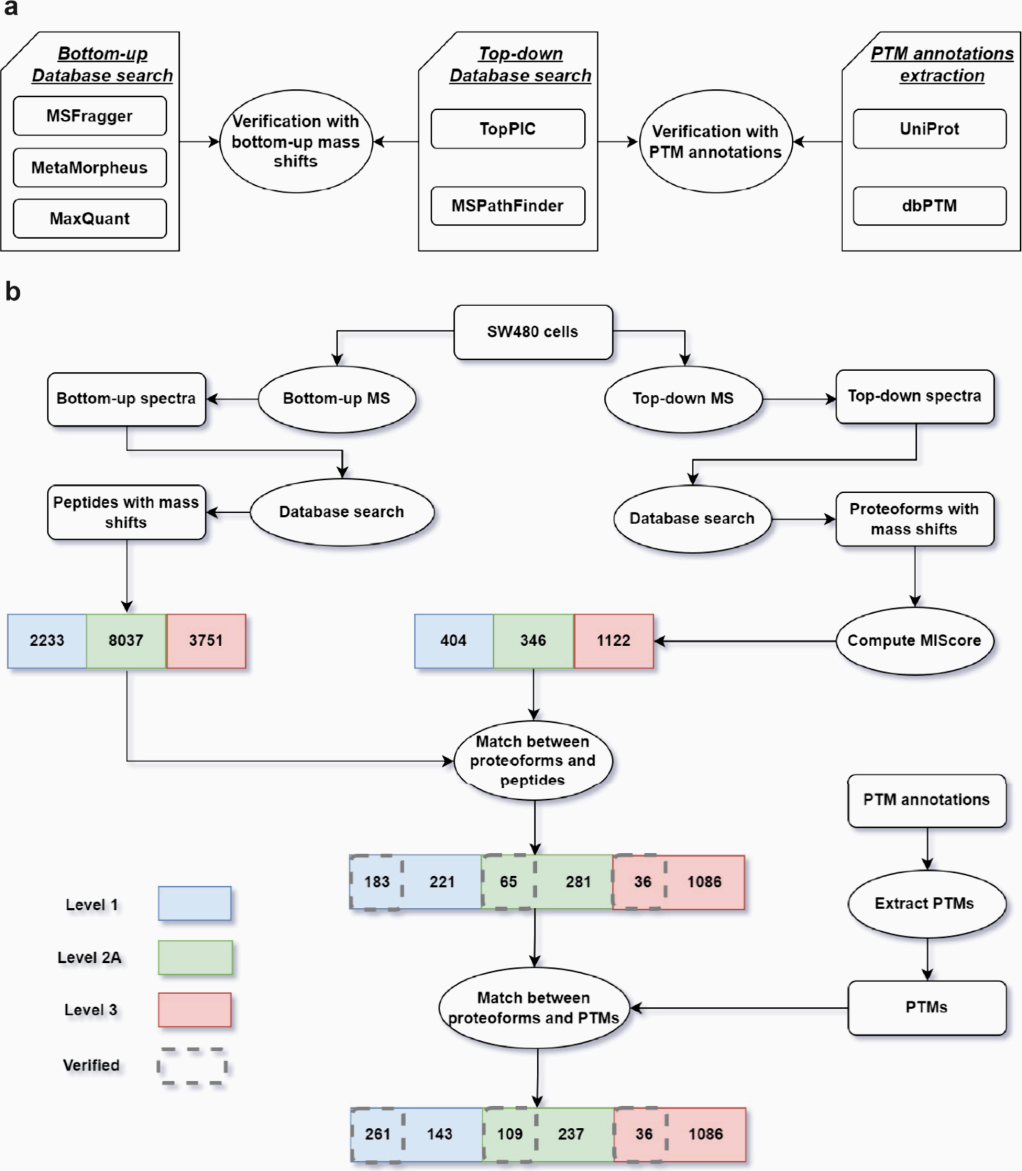
Overall scheme of the PTM-TBA pipeline for PTM identification, localization, and verification using top-down MS, bottom-up MS, and UniProt annotations. (a) Top-down MS data are first used for identifying mass shifts/PTMs in proteoforms, and then mass shifts/PTMs identified from bottom-up MS data and PTM annotations are employed to verify the mass shifts/PTMs identified from top-down MS data. MSFragger, MetaMorpheus, and MaxQuant are three options for peptide identification by bottom-up MS. TopPIC and MSPathFinder are two tools in the pipeline for proteoform identification by top-down MS. PTM annotations in UniProt and dbPTM are used for verifying PTM identifications. (b) The numbers of mass shift/PTM identifications are shown for the PTM-TBA analysis of the first replicate of the SW480 top-down and bottom-up MS data using MSFragger, TopPIC, and UniProt annotations. Mass shifts identified from bottom-up and top-down MS data are divided into three levels: level 1: mass shifts with identified PTM types and localized sites; level 2A: mass shifts with identified PTM types but without localized sites; level 3: mass shifts without PTM identification and localized sites.

Figure 1 illustrates the overall scheme of the PTM-TBA pipeline for PTM identification, localization, and verification with top-down MS, bottom-up MS data, and PTM annotations. Top-down MS data are first used for identifying mass shifts/PTMs in proteoforms, and then mass shifts/PTMs identified from bottom-up MS data and PTM annotations are employed to verify the mass shifts/PTMs identified from top-down MS data. MSFragger,^15^ MetaMorpheus,^43^ and MaxQuant^14^ are three software options for identifying mass shifts in peptides by database search using bottom-up MS data. TopPIC^27^ and MSPathFinder^24^ are two tools for identifying mass shifts from top-down MS data. PTM annotations in the UniProt knowledge base^32^ and dbPTM^46^ can be used for PTM verification. Figure 1b shows the analysis results of PTM-TBA for the first replicate of the SW480 bottom-up and top-down MS data. MSFragger^15^ was used for identifying mass shifts in peptides from the bottom-up MS data. After duplicated shifts were removed, mass shifts in peptides were categorized into three levels based on the five-level classification system for proteoform identifications proposed by Smith et al.^47^: level 1: mass shifts with identified PTM types and localized sites; level 2A: mass shifts with identified PTM types but without localized sites; level 3: mass shifts without PTM identification and localized sites. TopPIC^27^ was used to identified mass shifts from the top-down MS data. After duplicated shifts were removed, the identified mass shifts were also divided into three levels. The mass shifts in proteoforms were verified by matching them with mass shifts in peptides. PTM annotations were extracted from the UniProt knowledge base,^32^ and mass shifts in proteoforms were further confirmed by matching these PTM annotations to the mass shifts in proteoforms.

### 3.2 Mass Shifts Identified by Bottom-Up MS Using MSFragger

The sensitivity of PTM identification in bottom-up MS is affected by the selection of variable PTMs in a database search. Including a PTM as a variable PTM in database search will increase the identifications of the PTM and also increase the FDR of the PTM. To evaluate the performance of MSFragger with different settings of variable PTMs, we performed two rounds of database searches of the first replicate of the SW480 bottom-up MS data, which contained 99,427 MS/MS spectra. NTA and methionine oxidation were chosen as variable PTMs in the first round, and only NTA was set as the variable PTM in the second round (Section 2).

The first round of database search of MSFragger identified 72,241 PSMs, 28,141 peptides, and 3,825 proteins at 1% PSM-level, peptide-level, and protein-level FDRs, respectively. Of the 72,241 identified PSMs, 52,845 were from unmodified peptides and the remaining 19,396 were from modified peptides, which contained a total of 24,341 mass shifts (some identified peptides contained more than one mass shift/PTM).

**Figure 2:**
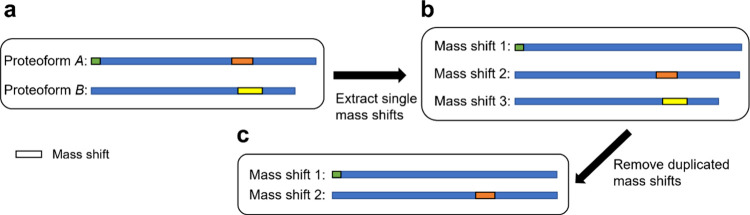
Illustration of duplication removal of mass shifts in proteoforms. (a) Proteoform identifications *A* and *B* are from the same protein. Proteoform *A* contains the whole protein sequence with two mass shifts (green and orange), and proteoform *B* is a truncated one with one mass shift (yellow). The colored parts show protein regions containing possible modification sites of the mass shift. (b) Three single mass shifts are extracted from the two proteoform identifications. (c) The orange and yellow mass shifts have similar shifts and their possible modification site regions overlap, so they are treated as duplicated ones and only one is kept.

**Figure 3:**
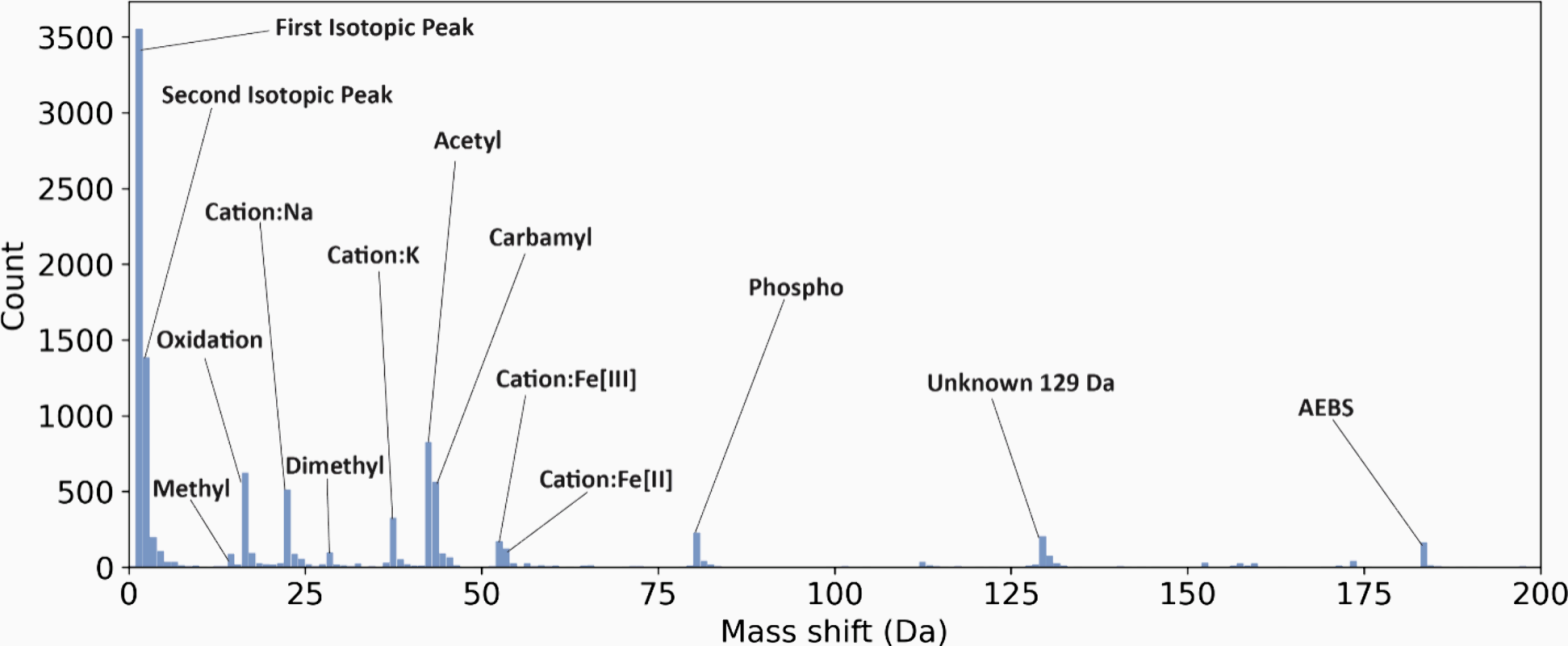
Histogram of mass shifts reported by MSFragger (round 1) from the first replicate of the SW480 bottom-up MS data in the range of [0, 200] Da.

A mass shift in a peptide may be reported in several identified PSMs (Figure 2a), so duplicated mass shifts need to be removed (Section 2). After duplicated mass shifts were removed, 14,073 mass shifts/PTMs sites were reported, including 754 NTA sites and 9567 mass shifts that were matched to the shift of a PTM. A total of 12 PTMs (Table S9) were identified with a high frequency (observed in >0.15% of all identified PSMs). Figure 3 shows the frequencies of mass shifts in the range of [0, 200] Da, in which high-frequency PTMs were labeled. The mass change of deamination PTM is 0.98 Da, which is similar to the isotopic error, so we removed it from the high-frequency PTM list. Using the 11 high-frequency PTMs, the 14,021 mass shifts were divided into three levels: 2233 level 1, including 721 NTA sites, 8037 level 2A, and 3751 level 3.

The second round of database search identified 72,199 PSMs, 28,169 peptides, and 3841 proteins with 1% FDR, which were similar to the numbers of identifications in the first round. We extracted 23,080 mass shifts from 19,264 identified PSMs with mass shifts or PTMs and finally obtained 13,545 mass shifts after removing duplicated ones. These mass shifts can be divided into the three levels: 309 level 1, including 726 NTA sites, 8103 level 2A, and 3133 level 3. When methionine oxidation was not set as a variable PTM, MSFragger identified only 274 oxidation sites, about 40.9% of the oxidation sites (670) reported in the first round, showing that setting a common PTM as a variable PTM can significantly increase the number of PTM sites identified by MSFragger. Besides methionine oxidation, MSFragger reported similar numbers of modification sites for other mass shifts in the two rounds (Figure S2).

We also assessed the reproducibility of mass shift identifications from bottom-up MS data using the three replicates of SW480 bottom-up MS data with MSFragger. The parameter settings were the same as those in the first round of the MSFragger database search. Of the 14,021 mass shifts identified from the first replicate, only 1340 (9.6%) were also reported from the other replicates (Figure S5a).

### 3.3 Comparison of MSFragger, MetaMorpheus, and MaxQuant

We compared the identification results of MSFragger (round 1), MetaMorpheus, and MaxQuant using the first replicate of the SW480 bottom-up MS data set (see Section 2). The open search strategy employed by MSFragger reported much more mass shifts (14,021 vs 4412 vs 885) than MetaMorpheus and MaxQuant. NTA, oxidation, and phosphorylation were set as variable PTMs in three rounds of database searches of MaxQuant (Methods), so we compared only the three PTMs identified by MSFragger and MaxQuant. MSFragger identified more NTA (721 vs 672 (round 1 of MaxQuant)), oxidation (670 vs 180 (round 2 of MaxQuant)), and phosphorylation (279 vs 266 (round 3 of MaxQuant)) sites than did MaxQuant (Table S10). Compared with MSFragger, MetaMorpheus reported more carbamylation sites (599 vs 475) and less dimethylation (73 vs 178), methylation (182 vs 380), and potassium adduct sites (40 vs 137), and the two tools reported similar numbers of the other high frequency modifications (Figure S3). The running times of MSFragger (round 1), MetaMorpheus, and MaxQuant (round 2) were 160, 59, and 166 min, respectively (Table S11).

### 3.4 Mass Shifts Identified by Top-Down MS Using TopPIC

We analyzed the first replicate of the SW480 top-down MS data, which contained 22,455 MS/MS spectra, against the GENCODE-SWISS database using TopPIC^27^ (version 1.6.0) (Section 2). TopPIC reported 2423 proteoforms with unexpected mass shifts or variable PTMs, including 57 histone proteoforms and 1407 proteoforms without any mass shifts/PTMs except for cysteine carbamidomethylation from 849 proteins with 1% proteoform-level FDR. The 57 histone proteoforms were excluded from downstream analysis because most of them contained multiple PTMs.

**Figure 4:**
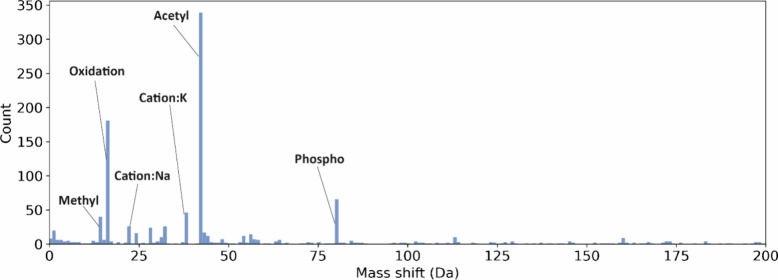
Histogram of mass shifts reported by TopPIC from the first replicate of the SW480 top-down MS data in the range of [0, 200] Da.

Similar to mass shifts in peptides, duplicated mass shifts and PTMs in identified proteoforms were removed. After removing duplicated mass shifts, we identified 320 NTA sites and 1552 other mass shifts in 1792 proteoforms, of which 80 contained both NTA and another mass shift. Figure 4 shows the distribution of the identified mass shifts in [0, 200] Da. The distribution of mass shifts in [−500, 500] Da is given in Figure S4. Oxidation, methylation, acetylation, and phosphorylation are the most frequently observed PTMs, which are consistent with those reported by bottom-up MS. Additionally, 125 mass shifts (8.1% of all reported mass shifts) have a shift around −43 Da, which can be explained by an incorrect assignment of carbamidomethylation on a cysteine with methylation.

The 1552 mass shifts were further matched to high-frequency PTMs identified from bottom-up MS to determine their PTM types. Because the mass shift (0.98 Da) of deamidation is similar to +1 Da errors in deconvoluted precursor masses, which are commonly observed, deamidation was excluded, and only the remaining 11 high-frequency PTMs in Table S9 were matched to the mass shifts reported by top-down MS. The PTM type of a mass shift was determined if (1) the mass shift was matched to the shift of a high-frequency PTM with an error tolerance of 0.1 Da (the error ±1.00235 Da was also allowed in the matching of the mass shift) and (2) the possible site of the mass shift contained at least one amino acid that can be modified by the PTM. Using the method, a total of 430 mass shifts were identified as high-frequency PTMs (Table S12), including 184 oxidation sites and 65 phosphorylation sites. For these 430 high-frequency PTMs, MIScore^29^ was used to localize their PTM sites, and 84 PTMs were confidently localized with a confidence score ≥0.6 (Table S12).

As a result, all the identified NTA sites, variable PTMs, and unexpected mass shifts were divided into 3 levels: 404 level 1 mass shifts with identified and localized PTM sites (including 320 NTA sites), 346 level 2A mass shifts with identified but not localized PTMs, and 1122 level 3 mass shifts without identified PTMs. The PTMs in level 1 (excluding NTA) and level 2A are listed in Table S12.

**Figure 5:**
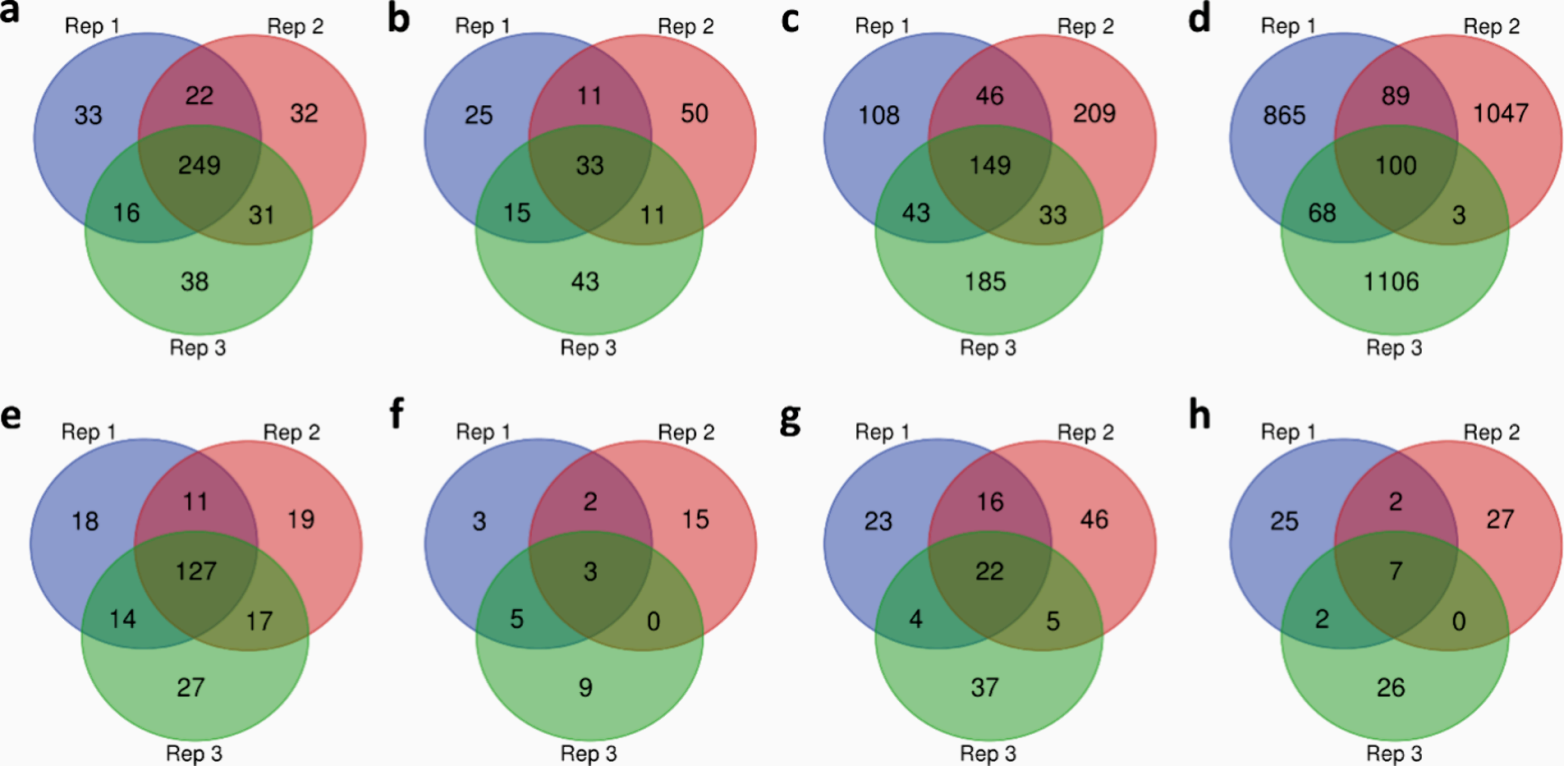
Comparison of mass shift identifications across the three replicates of SW480 top-down and bottom-up MS data. (a) NTA, (b) level 1 excluding NTA, (c) level 2A, and (d) level 3 mass shifts identified from the top-down MS data. (e) NTA, (f) level 1 excluding NTA, (g) level 2A, and (h) level 3 mass shifts identified from the top-down MS data and verified by the bottom-up MS data.

We evaluated the reproducibility of mass shift identifications using the three replicates of the SW480 top-down MS data. Of the 320 NTA sites, 84 level 1 excluding NTA, 346 level 2A, and 1122 level 3 mass shifts identified from the first replicate, 249 (77.8%) NTA, 33 (39.3%) level 1 excluding NTA, 149 (43.1%) level 2A, and 100 (8.9%) level 3 mass shifts were also identified in the other two replicates (Figure 5 a–d). The low reproducibility of level 3 mass shifts may indicate that they have a high FDR.

### 3.5 Comparison of TopPIC and MSPathFinder

**Figure 6:**
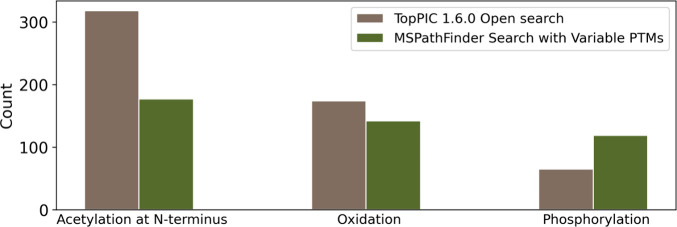
Comparison of PTMs identified by TopPIC and MSPathFinder from the first replicate of the SW480 top-down MS data.

Both MSPathFinder and TopPIC were used to analyze the first replicate of the SW480 top-down MS data for proteoform and mass shift identifications. NTA, oxidation, and phosphorylation were set as variable PTMs in MSPathFinder, so we compared only the three PTMs identified by the two tools. After removal of duplicated PTM identifications, MSPathFinder reported more phosphorylation sites (119 vs 65) and less NTA (177 vs 318) and oxidation sites (142 vs 174) than TopPIC (Figure 6). In addition, the running time of MSPathFinder was 3624 min, which was about 7.6 times of the running time of TopPIC (475 min) (Table S11). MSPathFinder reported 82 phosphorylation sites missed by TopPIC, while TopPIC reported 18 sites missed by MSPathFinder. For 49 of the 82 sites, TopPIC reported another proteoform with more matched fragment ions than the proteoform reported by MSPathFinder, showing that the 82 phosphorylation sites may have a large FDR.

### 3.6 Mass Shift and PTM Verification by Bottom-Up MS

We employed TopPIC and MSFragger (round 1) to identify mass shifts from the first replicate of the SW480 top-down and bottom-up MS data and then used mass shifts/PTMs identified from bottom-up MS data to verify mass shifts/PTMs reported from top-down MS data (Figure 1b). The running time of matching mass shifts/PTMs was less than 1 min (Table S11). The 1122 level 3 mass shifts identified by top-down MS were compared with all mass shifts (14,021 entries) reported from bottom-up MS by MSFragger, resulting in 36 matched mass shifts between identified proteoforms and peptides. The level 1 and level 2A PTMs reported by top-down MS were then compared with the PTMs in peptides reported by MSFragger (Section 2), and 183 Level 1 (including 170 NTA sites) and 65 level 2A PTMs were matched to those in peptides.

Next, we assessed the reproducibility of mass shift verification by bottom-up MS using three replicates of the SW480 data. Of 170 NTA sites, 13 level 1 excluding NTA, 68 level 2A, and 36 level 3 mass shifts identified from top-down MS data and verified by bottom-up MS data in the first replicate, 127 (74.7%) NTA, 3 (23.1%) level 1 excluding NTA, 22 (32.4%) level 2A, and 7 (19.4%) level 3 mass shifts were also identified and verified in the other two replicates (Figure 5e–h).

We also evaluated 4 combinations of software tools for identifying and verifying mass shifts using the first replicate of the SW480 data (Table S13), and the combination of MSFragger and TopPIC reported the largest number of mass shifts that were identified by top-down MS and verified by bottom-up MS.

### 3.7 PTM Verification Using Annotations

**Figure 7:**
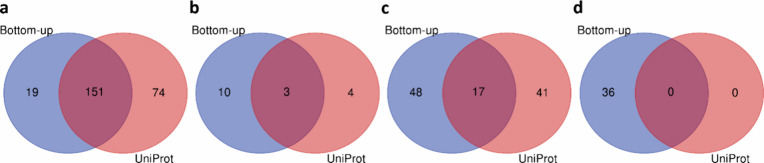
Comparison of mass shifts identified from the first replicate of the SW480 top-down MS data and verified by the first replicate of the SW480 bottom-up MS data and UniProt annotations. (a) NTA, (b) level 1 excluding NTA, (c) level 2A, (d) level 3.

The level 1 and 2A mass shifts in proteoforms identified by TopPIC from the first replicate of the SW480 top-down MS data were contrasted with all PTM annotations extracted from the UniProt database (Section 2). A total of 225 NTA sites in level 1, 7 other PTMs in level 1, and 58 PTMs in level 2A were confirmed by matching them to UniProt annotations. The verified PTMs included 5 methylation, 3 oxidation, 6 dimethylation, 232 acetylation (225 at the N-terminus), and 44 phosphorylation sites. Figure 7 illustrates the numbers of mass shifts verified by bottom-up MS only, UniProt annotations only, or both. Bottom-up MS and UniProt annotations provide complementary information for the mass shift verification. Of the 406 mass shifts verified by bottom-up MS or UniProt annotations, 119 mass shifts (29.3%) were verified by UniProt annotations only and 113 (27.8%) by bottom-up MS only.

The level 1 and Level 2A mass shifts identified by TopPIC from the first replicate of the SW480 top-down MS data were also compared with PTM annotations in the dbPTM^45^ database for verification. We downloaded and parsed annotations of acetylation, phosphorylation, methylation, oxidation, and hydroxylation from human proteins from the dbPTM database. Using these annotations, we verified 220 acetylation sites (210 at the N-terminus), 64 phosphorylation sites, 15 methylation sites, and 0 oxidation/hydroxylation sites (Table S14). By combining the annotations of UniProt and dbPTM, the numbers of verified acetylation, phosphorylation, and methylation sites were increased to 229, 65, and 15, respectively (Figure S6).

### 3.8 Evaluation of PTM-TBA with an Extended PTM List

We extended the PTM list in PTM-TBA to all the PTMs (1310 entries) stored in the UNIMOD database (version 07/18/2023) and tested PTM-TBA on the first replicate of the SW480 top-down MS data set. TopPIC identified 794 level 1 (including 316 NTA), 744 level 2A, and 484 level 3 mass shifts from identified PTMs. Furthermore, we verified 170 NTA, 24 level 1 excluding NTA, 94 level 2A, and 4 level 3 mass shifts using peptide identifications reported by MSFragger (round 1) and confirmed 225 NTA, 11 level 1 excluding NTA, and 59 level 2A mass shifts using UniProt annotations (Table S15). Compared with the analysis with the 11 high-frequency PTMs, more mass shifts were classified as level 1 (794 vs 404) and level 2A (744 vs 346) and fewer mass shifts were classified as level 3 (484 vs 1122) because including all UNIMOD PTMs into the PTM list increased the chance that a mass shift was matched to a PTM.

### 3.9 Evaluation on the Jurkat Data

To test the robustness of PTM-TBA, we evaluated its performance using the bottom-up and top-down data sets of Jurkat cells (Section 2). TopPIC and MSFragger (round 1) were used for mass shift identification from top-down and bottom-up MS data, respectively. All parameter settings were the same as those in the analysis of the SW480 data. TopPIC identified 1433 proteoforms from 472 proteins from the top-down MS data. We extracted 247 level 1 (including 233 NTA sites), 125 level 2A, and 672 level 3 mass shifts from the proteoform identifications. Using mass shifts identified from bottom-up MS and UniProt annotations, 138 level 1 and 30 level 2A mass shifts were confirmed (Table S16).

## 4 Conclusions and Discussion

We developed and evaluated PTM-TBA, a software pipeline for proteoform PTM identification, localization, and verification using top-down MS, bottom-up MS, and annotations. The pipeline successfully identified 814.5 ± 64.5 (40.7% of all reported mass shifts) high-frequency PTMs and localized 423 ± 19 PTM sites using SW480 top-down MS data. We also verified 399 ± 29 mass shifts in proteoforms using peptides identified by the SW480 bottom-up MS data and UniProt annotations.

Many PTMs identified from the top-down MS data remained unverified by bottom-up MS data. The main reason is that the sequence coverage of the proteins identified by bottom-up MS was low. The average sequence coverage of proteins identified from the first replicate of the SW480 bottom-up MS data by MSFragger (round 1) was 31% (Figure S7), so only a small fraction of PTMs identified by top-down MS could be covered and verified bottom-up peptide identifications. Increasing the protein sequence coverage in bottom-up MS is essential to improve the characterization of proteoforms with PTMs.

The reproducibility of the identified and verified mass shifts is low (Figure 5). On the SW480 data, the reproducibility of NTA identification and verification was above 74% and the reproducibility of the identification and verification of other mass shifts was below 45%. It is still a challenging problem to improve the reproducibility in top-down and bottom-up DDA-MS. Data-independent acquisition (DIA)-MS may be a potential method for improving the reproducibility in mass shift identification by top-down MS.

In this study, we focused on proteoforms with only one PTM and excluded proteoforms with multiple PTMs, such as histone proteoforms. The characterization of the proteoforms of multiple PTMs requires more advanced top-down MS methods that provide fragment ions with high proteoform sequence coverage as well as computational methods for automated multiple PTM identification and localization. Combining multiple fragmentation methods and including middle-down MS data can increase the proteoform sequence coverage of fragment ions, which is a future research direction.

Database searches with variable PTMs and the open search method are two complementary approaches for PTM identification in bottom-up and top-down proteomics. In a database search with variable PTMs, adding more variable PTMs will increase the sensitivity of PTM identification and significantly increase the running time of data analysis. Database searches with variable PTMs are suited for studies with a limited number of PTMs, which can be performed with a reasonable running time and high sensitivity for PTM identification. When many variable PTMs are considered, a multiple round approach can be adopted to reduce the running time of database search, in which the PTMs are divided into several groups, and only one group of PTMs is selected as variable PTMs in each round. Compared with a database search with variable PTMs, the open search strategy can identify unexpected PTMs with reasonable running time, but its sensitivity in PTM identification is lower than the variable PTM method due to the large search space of the open search method. In practice, these two methods can be combined to increase the sensitivity of PTM identification.

In PTM-TBA, a predefined PTM list is needed for PTM identification and verification. While including more PTMs in the list will increase the sensitivity of PTM identification, it may also increase the number of false PTM identifications. For example, the sum of the mass shifts of two common PTMs may be explained by an uncommon PTM. The sensitivity and accuracy of PTM identifications must be balanced when we choose the predefined PTM list in the pipeline.

Only a small number of PTM sites identified from the top-down MS data were verified by UniProt and dbPTM annotations, showing that the PTM annotations are incomplete. The human proteoform project^48^ aims to offer a comprehensive human proteoform annotation, which is essential for MS-based proteoform characterization. Proteoform and PTM annotations of other model species are also needed to increase the accuracy of the proteoform characterization.

## Data Availability

The code of PTM-TBA is available at https://github.com/wenronchen/PTM-TBA.

## Supporting Information Available

S-1: algorithm for removing duplicated mass shifts; S-2: extracting PTMs from UNIMOD; Figure S1: greedy algorithm for removing duplicated mass shifts; Figure S2: histogram of mass shifts reported by MS-Fragger (round 2) from the first replicate of the SW480 bottom-up MS data in the range of [−500, 500] Da; Figure S3: comparison of high-frequency PTMs identified by MSFragger and MetaMorpheus from the first replicate of the SW480 bottom-up MS data; Figure S4: histogram of mass shifts reported by TopPIC from the first replicate of the SW480 top-down MS data in the range of [−500, 500] Da; Figure S5: comparison of mass shifts identified and verified by the three replicates of the SW480 top-down and bottom-up MS data; Figure S6: comparison of NTA, phosphorylation, and methylation sites identified from the first replicate of the SW480 top-down MS data and verified by UniProt and dbPTM annotations; Figure S7: distribution of protein sequence coverage of peptides identified by MSFragger (round 1) from the first replicate of the SW480 bottom-up MS data; Table S1: parameter settings of MSFragger; Table S3: parameter settings of MetaMorpheus; Table S4: parameter settings of MaxQuant; Table S5: parameter settings of TopFD; Table S6: parameter settings of TopPIC; Table S7: parameter settings of ProMex; Table S8: parameter settings of MSPathFinder; Table S9: high-frequency PTMs reported by MSFragger (round 1) from the first replicate of the SW480 bottom-up MS data; Table S10: comparison of MSFragger, MetaMorpheus, and MaxQuant for mass shift identification using the first replicate of the SW480 bottom-up MS data; Table S11: running times of software tools and matching functions in PTM-TBA for analyzing the first replicate of the SW480 data; Table S13: comparison of several combinations of software tools for mass shift identification and verification using the first replicate of SW480 data; Table S14: numbers of annotated PTM sites downloaded from the dbPTM database and PTM sites identified from the first replicate of the SW480 top-down MS data and verified by dbPTM annotations (PDF)
Table S2: complete list of modifications used for G-PTM-D (XLSX)
Table S12: complete list of mass shifts identified from the first replicate of SW480 top-down MS data and verified by the first replicate of SW480 bottom-up MS data and annotations (XLSX)
Table S15: complete list of mass shifts identified by the first replicate of the SW480 top-down MS data and verified by the first replicate of the SW480 bottom-up MS data and UniProt annotations using all PTMs in the UNIMOD database (XLSX)
Table S16: complete list of mass shifts identified from the Jurkat top-down MS data and verified by the Jurkat bottom-up MS data and UniProt annotations (XLSX)
