# Supplementary material for: Characterization of Proteoform Post-Translational Modifications by Top-Down and Bottom-Up Mass Spectrometry in Conjunction with Annotations
Source: J Proteome Res. 2023 Sep 20;22(10):3178–89. doi: 10.1021/acs.jproteome.3c00207 (PMC10563160; doi:10.1021/acs.jproteome.3c00207)
Supplement: Supplementary file 1 — pr3c00207_si_001.pdf [file pr3c00207_si_001.pdf]

Characterization of proteoform post-translational modifications by top-down and bottom-up mass spectrometry in conjunction with annotations

Wenrong Chen[1], Zhengming Ding[2], Yong Zang[3,4], and Xiaowen Liu[5,6*]

[1]Department of BioHealth Informatics, Indiana University-Purdue University Indianapolis, Indianapolis, IN 46202, USA, [2]Department of Computer Science, Tulane School of Science and Engineering, Tulane University, New Orleans, LA 70118, USA, [3]Department of Biostatics and Health Data Sciences, Indiana University School of Medicine, Indianapolis, IN 46202, USA, [4]Center for Computational Biology and Bioinformatics, Indiana University School of Medicine, Indianapolis, IN 46202, USA [5]Tulane Center for Biomedical Informatics and Genomics, Tulane University, New Orleans, LA 70112, USA, [6]Deming Department of Medicine, Tulane University, New Orleans, LA 70112, USA

*Corresponding author: 1441 Canal St, New Orleans, LA 70112, xwliu@tulane.edu

Table of Contents

Materials

S-1: An algorithm for removing duplicated mass shifts

S-2: Extracting PTMs from UNIMOD

Figures

Figure S1: A greedy algorithm for removing duplicated mass shifts

Figure S2. A histogram of mass shifts reported by MS-Fragger (round 2) from the first replicate of the SW480 bottom-up MS data in the range [-500, 500] Da

Figure S3: Comparison of high-frequency PTMs identified by MS-Fragger and MetaMorpheus from the first replicate of the SW480 bottom-up MS data

Figure S4: A histogram of mass shifts reported by TopPIC from the first replicate of the SW480 top-down MS data in the range [-500, 500] Da

Figure S5: Comparison of mass shifts identified and verified by the three replicates of the SW480 top-down and bottom-up MS data

Figure S6: Comparison of NTA, phosphorylation, and methylation sites identified from the first replicate of the SW480 top-down MS data and verified by UniProt and dbPTM annotations

Figure S7: The distribution of protein sequence coverage of peptides identified by MS-Fragger (round 1) from the first replicate of the SW480 bottom-up MS data

Tables

Table S1: Parameter settings of MS-Fragger

Table S2: The complete list of modifications used for G-PTM-D (Table_S2.xlsx)

Table S3: Parameter settings of MetaMorpheus

Table S4: Parameter settings of MaxQuant

Table S5: Parameter settings of TopFD

Table S6: Parameter settings of TopPIC

Table S7: Parameter settings of ProMex

Table S8: Parameter settings of MSPathFinder

Table S9: High frequency PTMs reported by MS-Fragger (round 1) from the first replicate of the SW480 bottom-up MS data

Table S10: Comparison of MS-Fragger, MetaMorpheus and MaxQuant for mass shift identification using the first replicate of the SW480 bottom-up MS data

Table S11: Running times of software tools and matching functions in PTM-TBA for analyzing the first replicate of the SW480 data

Table S12: The complete list of mass shifts identified from the first replicate of SW480 top-down MS data and verified by the first replicate of SW480 bottom-up MS data and annotations (Table_S12.xlsx)

Table S13: Comparison of several combinations of software tools for mass shifts identification and verification using the first replicate of SW480 data

Table S14: Numbers of annotated PTM sites downloaded from the dbPTM database and PTM sites identified from the first replicate of the SW480 top-down MS data and verified by dbPTM annotations

Table S15: The complete list of mass shifts identified by the first replicate of the SW480 top-down MS data and verified by the first replicate of the SW480 bottom-up MS data and UniProt annotations using all PTMs in the UNIMOD database (Table_S15.xlsx)

Table S16: The complete list of mass shifts identified from the Jurkat top-down MS data and verified by the Jurkat bottom-up MS data and UniProt annotations (Table_S16.xlsx)

S-1: An algorithm for removing duplicated mass shifts

To remove duplicated mass shifts, we first group mass shifts reported from top-down or bottom-up MS data into clusters and then remove duplicated mass shifts in each cluster. In the clustering step, two mass shifts [m_1, p_1, a_1, b_1] and [m_2, p_2, a_2, b_2] are added to the same cluster if p_1 and p_2 are the same and the difference between m_1 and m_2 is smaller than an error tolerance. A greedy algorithm is used to remove duplicated mass shifts in the same cluster with the objective of reporting a set of non-duplicated mass shifts and maximizing the number of mass shifts (Fig. S1). We sort all mass shifts in a cluster from a protein in the increasing order of the left boundary. Let $L = S_1, S_2, \ldots, S_n$ be the sorted mass shifts of the cluster. We compare the boundaries (a_1, b_1) of mass shift S_1 with the boundaries (a_2, b_2) of S_2 to remove duplicated ones. There are three cases. Case 1: $b_1 \leq a_2$, that is, S_1 and S_2 do not overlap (Step 4 in Fig. S1). In this case, S_1 is removed from the mass shift list L and added to the result list R. Case 2: $a_2 < b_1 < b_2$, that is, S_1 and S_2 partially overlap (Step 6 in Fig. S1). In this case, S_2 is removed from the list L. Case 3, $b_1 \geq b_2$, that is, S_1 fully covers S_2 (Step 8 in Fig. S1). In this case, S_1 is removed from the list L. The comparison step is repeated for the first two mass shifts in L until only one mass shift remains in the list. Finally, the last remaining mass shift is added to the result list R.

S-2: Extracting PTMs from UNIMOD

All PTMs in the UNIMOD database (version 07/18/2023) [45] were downloaded in the text format. A Python script in PTM-TBA was used to extract the UNIMOD ID, PSI-MS name, monoisotopic mass, modified amino acid residues, mortification positions (N-terminal, C-terminal, or any) of each UNIMOD PTM from the downloaded text file.

Figures

Algorithm 1 Greedy algorithm for removing duplicated mass shifts

Input L: A list of mass shifts sorted in the increasing order of the left boundary.

Output R: A list of non-overlapping mass shifts.

while L contains ≥ 2 mass shifts **do**
    Let S_1, S_2 be the first two mass shifts in L, and (a_1,b_1),(a_2,b_2) be
    the boundaries of S_1 and S_2, respectively
    if $b_1 \leq a_2$ **then** # Case 1: S_1 and S_2 do not overlap
        remove S_1 from L and **add** S_1 to R
    else if $a_2 < b_1 < b_2$ **then** # Case 2: S_1 and S_2 partially overlap
        remove S_2 from L
    else # Case 3: S_1 fully covers S_2
        remove S_1 from L
    end if
end while
add the last mass shift in L to R
return R

Figure S1. A greedy algorithm for removing duplicated mass shifts

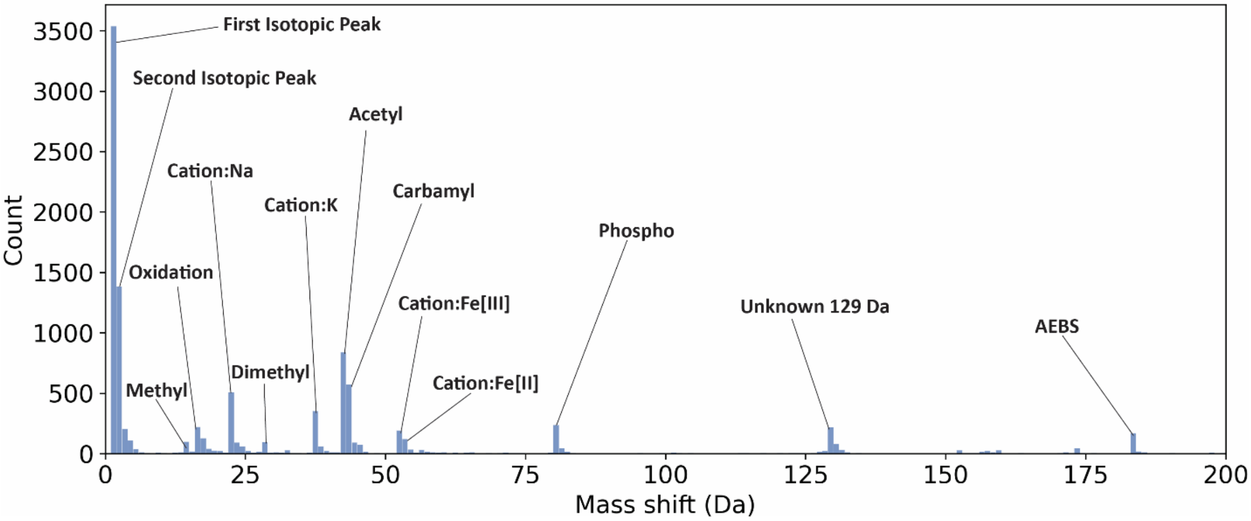

Figure S2. A histogram of mass shifts reported by MS-Fragger (round 2) from the first replicate of the SW480 bottom-up MS data in the range [-500, 500] Da.

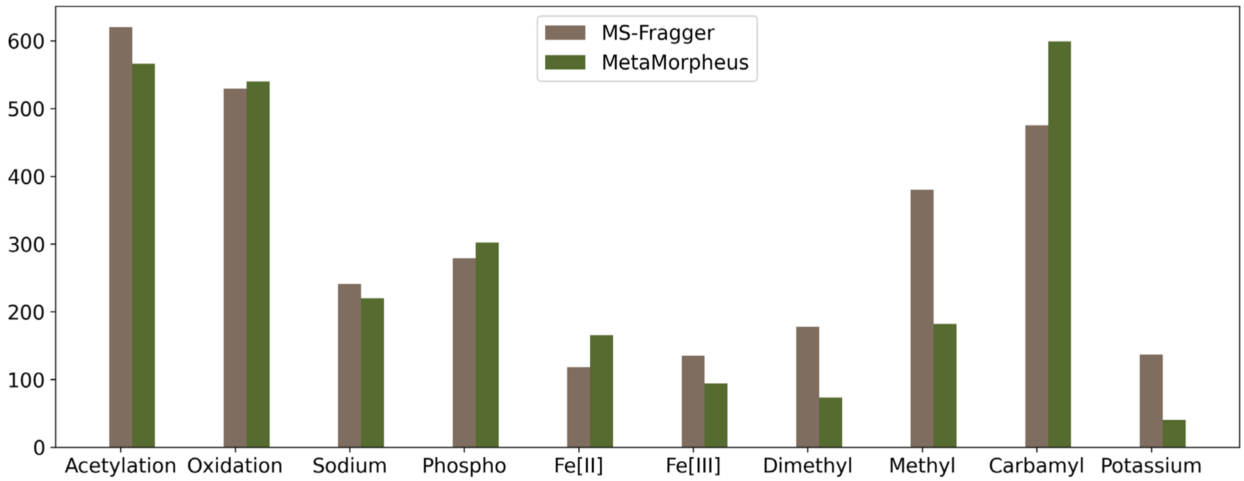

Figure S3. Comparison of high-frequency PTMs identified by MS-Fragger and MetaMorpheus from the first replicate of the SW480 bottom-up MS data. Aminoethylbenzenesulfonylation (AEBS) is not included in the comparison because it was not a variable PTM in the database search of MetaMorpheus.

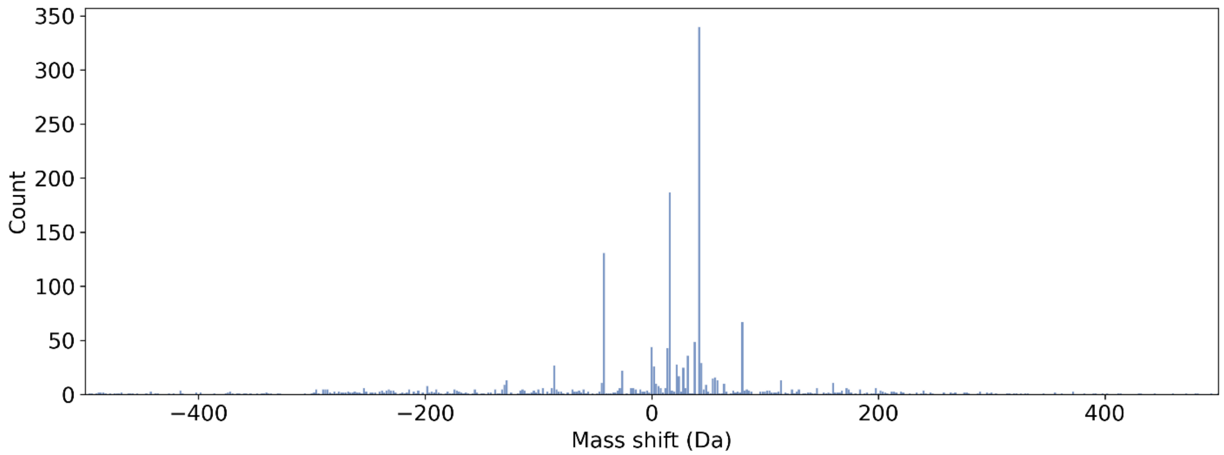

Figure S4. A histogram of mass shifts reported by TopPIC from the first replicate of the SW480 top-down MS data in the range [-500, 500] Da.

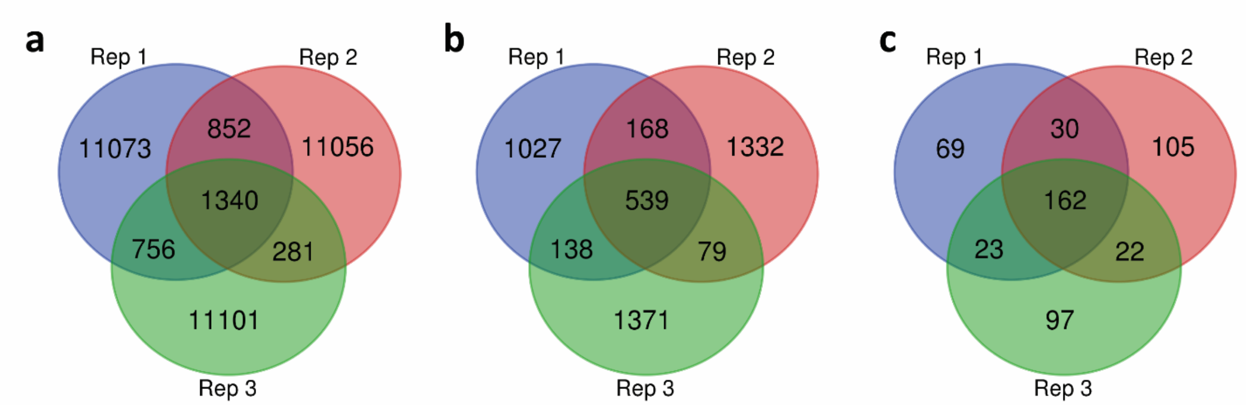

Figure S5. Comparison of mass shifts identified and verified by the three replicates of the SW480 top-down and bottom-up MS data. (a) Mass shifts identified from bottom-up MS data; (b) mass shifts identified from top-down MS data; (c) mass shifts identified by top-down MS data and verified by bottom-up MS data.

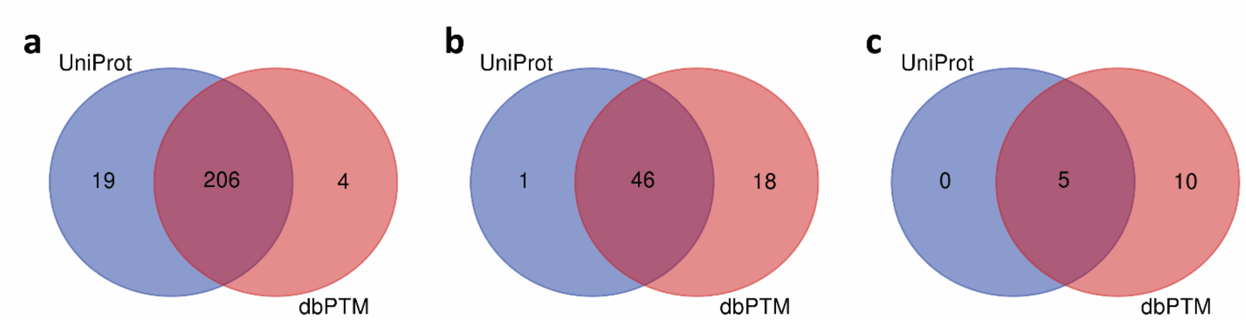

Figure S6. Comparison of NTA, phosphorylation, and methylation sites identified from the first replicate of the SW480 top-down MS data and verified by UniProt and dbPTM annotations: (a) NTA, (b) phosphorylation and (c) methylation.

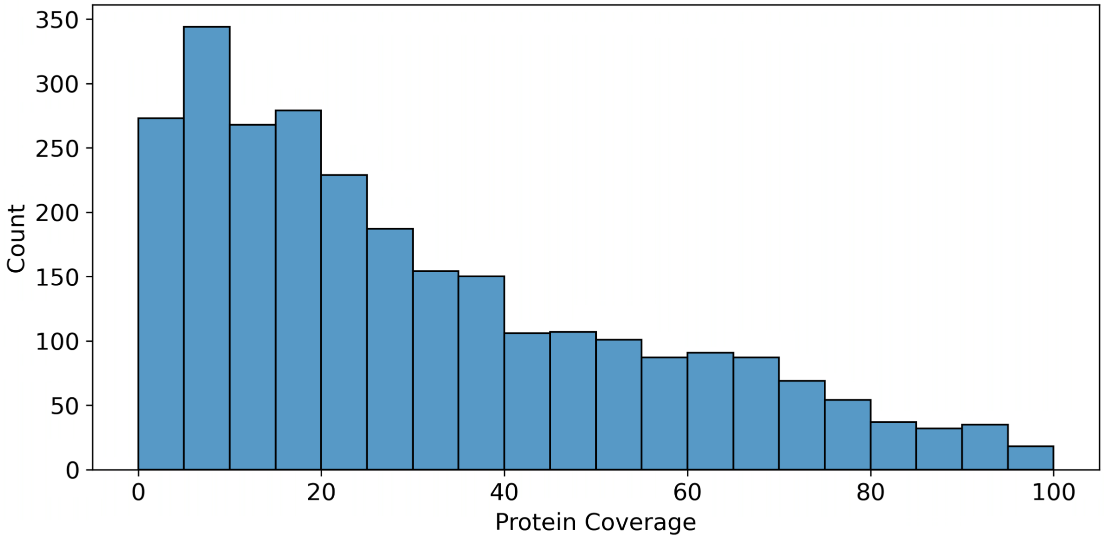

Figure S7. The distribution of protein sequence coverage of peptides identified by MS-Fragger (round 1) from the first replicate of the SW480 bottom-up MS data.

Tables

Table S1. Parameter settings of MS-Fragger.

MS-Fragger	
precursor_mass_lower	-150 Da
precursor_mass_upper	500 Da
precursor_true_tolerance	20 ppm
fragment_mass_tolerance	20 ppm
calibrate_mass	ON and find optimal parameters
Isotope_error	0
precursor_mass_mode	corrected
max_variable_mods_per_peptide	3
max_variable_mods_combinations	5000
digest_min_length	7
digest_max_length	50
digest_mass_range	500.0 Da to 5000.0 Da
Variable PTMs for round 1	15.994900 M 3<br>42.010600 [^ 1
Variable PTMs for round 2	42.010600 [^ 1

Table S3. Parameter settings of MetaMorpheus.

MetaMorpheus	
Search Mode	Classic Search
In-silico Digestion Parameters	
Generate target proteins	
Generate decoy proteins	
Generate reversed decoys	
Max Missed Cleavages	2
Initiator Methionine	Variable
Max Modification Isoforms	1024
Min Peptide Length	7
Max Peptide Length	50
Max mods per peptide	3
Fragment Ion Search Parameters	
Dissociation Type	HCD
Max Fragment Mass (Da)	30000
Ambiguity Parameters	Report PSM ambiguity

Table S4. Parameter settings of MaxQuant.

MaxQuant	
PSM FDR	0.01
Protein FDR	0.01
Min. peptide Length	7
Min. score for unmodified peptides	0
Min. score for modified peptides	40
Min. delta score for unmodified peptides	0
Min. delta score for unmodified peptides	6
Max. peptide mass [Da]	5000
Min. peptide length for unspecific search	8
Max. peptide length for unspecific search	25
Max mods in site table	3
MS/MS tol. (Unknown)	20 ppm
MS/MS deisotoping tolerance (Unknown)	7 ppm
Variable modifications for round 1	Acetyl (Protein N-term)
Variable modifications for round 2	Acetyl (Protein N-term)<br>Oxidation (M)
Variable modifications for round 3	Acetyl (Protein N-term)<br>Phospho (STY)

Table S5. Parameter settings of TopFD.

TopFD	
Maximum charge	30
Maximum monoisotopic mass	70000 Dalton
Peak error tolerance	0.02 m/z
MS1 signal/noise ratio	1
Thread number	15
Precursor window size	3 m/z

Table S6. Parameter settings of TopPIC.

TopPIC	
Search type	TARGET+DECOY
Fixed modification	Carbamidomethylation 57.0215 C
Allowed N-terminal forms	NONE,NME,NME_ACETYLATION,M_ACETYLATION
Maximum number of variable modifications	1
Variable modifications	Oxidation (M)<br>Phospho (STY)
Maximum number of unexpected modifications	1
Maximum mass shift of modifications	500 Da
Minimum mass shift of modifications	-500 Da
Spectrum-level cutoff type	FDR
Spectrum-level cutoff value	0.01
Proteoform-level cutoff type	FDR
Proteoform-level cutoff value	0.01
Error tolerance for matching masses	10 ppm
Error tolerance for identifying PrSM clusters	1.2 Da
Localization with MIScore	True

Table S7. Parameter settings of ProMex.

TopPIC	
minCharge	2
maxCharge	60
minMass	3000
maxMass	50000
maxThreads	15

Table S8. Parameter settings of MSPathFinder.

MSPathFinder	
Internal Cleavage Mode	SingleInternalCleavage
Maximum Threads	15
TDA Mode	Target+Decoy
Precursor Ion Tolerance	10 ppm
Product Ion Tolerance	10 ppm
Min Sequence Length	21
Max Sequence Length	300
Min Precursor Ion Charge	2
Max Precursor Ion Charge	50
Min Product Ion Charge	1
Max Product Ion Charge	20
Min Sequence Mass	3000
Max Sequence Mass	50000
Max Dynamic Mods Per Sequence	1
Static Modifications	Carbamidomethyl (C), any position
Dynamic Modifications	Oxidation (M), any position<br>Phospho (STY), any position<br>Acetyl (any amino acid), Protein N-terminal

Table S9. High frequency PTMs reported by MS-Fragger (round 1) from the first replicate of the SW480 bottom-up MS data. Commonly modified residues and uncommonly modified residues are obtained from the UNIMOD database.

PSI-MS Name	Description	Modified residues (common)	Modified residues (uncommon)	Mass shift (Da)	% PSMs
Carbamyl	Carbamylation	K	RCMSTY	43.005814	1.10
Cation: Na	Sodium adduct	DE	-	21.981943	0.96
Cation: K	Replacement of proton by potassium	-	ED	37.955882	0.68
Phospho	Phosphorylation	TSY	DHCRKE	79.966331	0.58
AEBS	Aminoethylbenzene - sulfonylation	-	HKSY	183.035399	0.37
Cation: Fe [III]	Replacement of 3 protons by iron	-	DE	52.911464	0.34
Methyl	Methylation	-	CHKNQRILE DST	14.01565	0.28
Cation: Fe [II]	Replacement of 2 protons by iron	-	DE	53.919289	0.22
Dimethyl	di-Methylation	-	KRNP	28.0313	0.22
Acetyl	Acetylation	K	CSTYHR	42.010565	0.19
Oxidation	Oxidation or Hydroxylation	MWH	DKNPFYRC GUEILQSTV	15.994915	0.16

Table S10. Comparison of MS-Fragger, MetaMorpheus and MaxQuant for mass shift identification using the first replicate of the SW480 bottom-up MS data

	# Peptides	# Proteins	# Mass shifts	# NTA	# Oxidation	# Phospho
MS-Fragger	28,141	3,825	14,021	721	670	279
MetaMorpheus	25,562	3,729	4,412	468	540	302
MaxQuant (round 1)	24,381	3,597	672	672	-	-
MaxQuant (round 2)	24,553	3,595	855	675	180	-
MaxQuant (round 3)	24,521	3,593	945	679	-	266

Table S11. Running times of software tools and matching functions in PTM-TBA for analyzing the first replicate of the SW480 data

Program	Operating system	CPU	#Threads	Running time (min)
TopPIC	Linux	Intel(R) Xeon(R) CPU E5-2637 v4 @ 3.50GHz	15	475
MSPathFinder	Linux	Intel(R) Xeon(R) CPU E5-2637 v4 @ 3.50GHz	15	3,624
MS-Fragger (round 1)	Linux	Intel(R) Core(TM) i7-11700T @ 1.40GHz	1	160
MetaMorpheus	Linux	Intel(R) Xeon(R) CPU E5-2637 v4 @ 3.50GHz	15	59
MaxQuant (round 2)	Windows	Intel(R) Core(TM) i7-10700 @ 2.90GHz	4	166
Match between top-down and bottom-up mass shifts	Linux	Intel(R) Core(TM) i7-11700T @ 1.40GHz	1	<1
Match between mass shifts and annotations	Linux	Intel(R) Core(TM) i7-11700T @ 1.40GHz	1	<1

Table S13. Table S14: Comparison of several combinations of software tools for mass shifts identification and verification using the first replicate of SW480 data

Software tools		# Mass shifts verified		
Bottom-up	Top-down	Level 1	Level 2A	Level 3
MS-Fragger (round 1)	TopPIC	183	65	36
MS-Fragger (round 2)	TopPIC	180	62	36
MetaMorpheus	TopPIC	114	30	-
MS-Fragger (round 1)	MSPathFinder	35	-	-

Table S14. Numbers of annotated PTM sites downloaded from the dbPTM database and PTM sites identified from the first replicate of the SW480 top-down MS data and verified by dbPTM annotations

PTM type	# Total annotated sites	# Sites identified by top-down MS and verified by dbPTM
Acetylation	46,114	220
Phosphorylation	506,547	64
Methylation	8,744	15
Oxidation	38	0
Hydroxylation	483	0
